# Upadacitinib Versus Filgotinib in Ulcerative Colitis: Is the Evidence Sufficient to Inform Treatment Decisions?

**DOI:** 10.1002/ueg2.12709

**Published:** 2024-11-13

**Authors:** Sudheer Kumar Vuyyuru, Yuhong Yuan, Vipul Jairath

**Affiliations:** ^1^ Department of Medicine, Division of Gastroenterology Western University London Canada; ^2^ Lawson Health Research Institute Western University London Canada; ^3^ Department of Epidemiology and Biostatistics Western University London Canada

Several monoclonal antibodies have been approved for the treatment of moderate to severe ulcerative colitis (UC) including anti‐tumour necrosis factor agents, anti‐integrins as well as those targeting the IL‐12–23 and IL‐23 pathways [1]. Despite these available therapies, approximately 20% of patients experience primary non‐response [2]. Monoclonal antibodies also have other limitations including the development of anti‐drug antibodies and need for parenteral administration. Janus kinase (JAK) inhibitors (JAKi) are a group of oral small molecules that have been approved for moderate‐to‐severe UC and have helped to address some of the unmet needs of biological therapy and have expanded the therapeutic armamentarium for UC. JAKi hold several advantages over monoclonal antibodies which include rapid onset of action, rapid absorption after oral administration with short half‐life, ability to target a broad range of cytokines, lack of immunogenicity and have more predictable pharmacokinetics [3].

Tofacitinib was the first JAKi approved for the management of moderate to severely active UC including both biologic naïve patients as well as those who had failed prior biologics. This was subsequently followed by the approval of filgotinib (only approved by European Medical Agency but not by Food and Drug Administration [FDA]) and upadacitinib for the management of UC [4, 5]. JAKi have variable selectivity and affinity towards JAK, which hypothetically may be associated with differences in efficacy and safety. Tofacitinib is a pan JAKi with increased selectivity for JAK1 and JAK3, whereas upadacitinib and filgotinib are JAK1 selective inhibitors. However, in the absence of direct head‐to‐head trials of JAKi, which are highly unlikely, indirect comparisons may provide further information on the relative efficacy and safety of different JAKi. A recent network meta‐analysis ranked upadacitinib as superior to tofacitinib and filgotinib for efficacy but there no difference was found in adverse events among these agents [6]. Some real‐world studies have also compared the efficacy of upadacitinib to tofacitinib. In a recent study by Lu et al, in which individual patient data from the SELECTION trial for filgotinib 200 mg were weighted to match average baseline characteristics of active treatment and placebo arms in comparator trials [7]. In this study filgotinib demonstrated similar clinical remission, clinical response and endoscopic improvement compared to tofacitinib in both biologic naïve and experienced UC patients. However, data comparing efficacy and safety of filgotinib and upadacitinib are scarce.

In the recent issue of United European Gastroenterology Journal, Nogami and colleagues conducted a retrospective, real‐world observational study comparing the efficacy and safety of filgotinib and upadacitinib in 168 patients treated at three tertiary care centres in Japan, with a median follow up of approximately 6 months. The authors performed multivariable analysis and 1:1 propensity score matching (PSM) sensitivity analysis to balance baseline characteristics (sex, age, partial Mayo score, disease extent, concomitant medications, previous biologics and tofacitinib use, laboratory data, and smoking) in 104 patients. The authors demonstrated that upadacitinib was associated with significantly higher risk of clinical response (adjusted risk ratio [RR] 1.40, 95% CI [1.09–1.80]) and clinical remission (adjusted RR 1.54, 95% CI, [1.16–2.05]) at week 8 compared to filgotinib. Similar findings were observed on PSM analysis. On subgroup analysis based on prior tofacitinib exposure, there was no difference between upadacitinib and filgotinib for clinical remission (adjusted RR 1.77, 95% CI [0.84–3.73]) in tofacitinib exposed patients. However, upadacitinib was associated with greater clinical remission in prior biologic exposed (adjusted RR 2.06, 95% CI [1.36–3.13]) patients. This could be because of small number of patients with prior tofacitinib exposure. On analysis of safety outcomes, upadacitinib was associated with increased adverse events (45.7% vs. 24.5%) compared filgotinib most common of which were skin rash (17.1% vs. 3.1%) and acne (10% vs. 2%) but notably, no major cardiovascular events, venous thrombosis were observed with either filgotinib or upadacitinib.

The author team are to be congratulated on providing important new data to help inform clinical practice. The study offers novel insights on comparative efficacy and safety of upadacitinib and filgotinib. Inherent limitations include the retrospective design and relatively small sample size. The therapeutic choice of agent can be influenced by several factors which are difficult to control and can introduce risk of selection bias as well as residual confounding. In an attempt to overcome this, the authors performed propensity matched analysis adjusted for potential confounding factors showed consistent results supporting superior efficacy of upadacitinib. Additionally, mucosal healing was not assessed in this study which would also add valuable information.

To conclude these available real‐world data suggest that upadacitinib may have superior efficacy compared to filgotinib, especially in patients with prior biologic exposure although higher rates of AEs. However, without well‐designed prospective comparative clinical trials, firm conclusions cannot be drawn. Future studies with larger sample size are needed to evaluate comparative efficacy of these agents in patient with prior exposure to tofacitinib.

## Disclosures

S.K.V. has received consulting fee from Alimentiv. Y.Y. has none to disclose. V.J. received has received consulting/advisory board fees from AbbVie, Alimentiv, Arena pharmaceuticals, Asahi Kasei Pharma, Asieris, Astra Zeneca, Avoro Capital, Bristol Myers Squibb, Celltrion, Eli Lilly, Endpoint Health, Enthera, Ferring, Flagship Pioneering, Fresenius Kabi, Galapagos, Gilde Healthcare, GlaxoSmithKline, Genentech, Gilead, Innomar, JAMP, Janssen, Merck, Metacrine, Mylan, MRM Health, Pandion, Pendopharm, Pfizer, Protagonist, Prometheus Biosciences, Reistone Biopharma, Roche, Roivant, Sandoz, Second Genome, Sorriso, Synedgen, Takeda, TD Securities, Teva, Topivert, Ventyx, Vividion; speaker's fees from, Abbvie, Ferring, Bristol Myers Squibb, Galapagos, Janssen Pfizer Shire, Takeda, Fresenius Kabi.

## Data Availability

Data sharing is not applicable to this article as no new data were created or analysed in this study.
